# Discernment between candidate mechanisms for KRAS G13D colorectal cancer sensitivity to EGFR inhibitors

**DOI:** 10.1186/s12964-020-00645-3

**Published:** 2020-11-05

**Authors:** Thomas McFall, Noah K. Schomburg, Kent L. Rossman, Edward C. Stites

**Affiliations:** 1 Integrative Biology Laboratory, Salk Institute for Biological Studies, 10010 N. Torrey Pines Rd, La Jolla, CA 92037 USA; 2 Department of Surgery and the Lineberger Comprehensive Cancer Center, University of North Carolina School of Medicine, Chapel Hill, North Carolina 27599 USA

**Keywords:** KRAS, GTPase, EGFR, Cancer, Targeted therapy

## Abstract

Phase three clinical trial evidence suggests that colorectal cancers with the KRAS G13D mutation may benefit from EGFR inhibitors, like cetuximab, in contrast to the other most common KRAS mutations. A mechanism to explain why this mutation behaves differently from other KRAS mutations had long been lacking. Two recent studies have reproduced KRAS G13D specific sensitivity to cetuximab in cellular models, and both have implicated the tumor suppressor NF1 as a critical variable in determining sensitivity and resistance. One study proposes a mechanism that focuses on the inhibition of active, GTP-bound wild-type RAS, which is proposed to occur to a greater extent in KRAS G13D tumors due to the inability of KRAS G13D to bind NF1 well. The other study suggests NF1 can convert GTP-bound KRAS G13D to inactive, GDP-bound KRAS G13D. Here, we report an inability to reproduce cellular and biophysical studies that suggested NF1 has strong GTPase activity on KRAS G13D. We also report additional data that further suggests only WT RAS-GTP levels are reduced with EGFR inhibition and that KRAS G13D is impaired in binding to NF1. These new experiments further support a mechanism in which cetuximab inhibits wild-type (HRAS and NRAS) signals in KRAS G13D colorectal cancers.

**Video Abstract**

**Supplementary Information:**

The online version contains supplementary material available at 10.1186/s12964-020-00645-3.

## Background

The Epidermal Growth Factor Receptor (EGFR) proto-oncogene has proven to be a valuable target in multiple types of cancer, including colorectal, lung, and head and neck cancer [1]. Upon ligand binding, EGFR dimerizes and its kinase domain becomes catalytically active [2]. Trans-phosphorylation of tyrosine residues on EGFR allows for the recruitment of proteins that harbor Src Homology 2 (SH2) and/or Protein Tyrosine Binding (PTB) domains, both of which are capable of binding to phospho-tyrosine residues [3]. These protein-protein interactions lead to the activation of multiple signaling pathways, including the RAS/RAF/MEK/ERK Mitogen Activated Protein Kinase (MAPK) cascade [4].

Mutations to members of the EGFR-RAS signaling pathway that result in a net activation of this pathway are common in a wide-variety of cancers. Mutant forms of many of these proteins, including EGFR and BRAF, have proven to be valuable drug targets for which small molecule inhibitors have been developed and received FDA-approval [5, 6]. Mutations within this pathway can also cause resistance to treatments that target this pathway. For example, the presence of a KRAS mutation has long been recognized as a marker of resistance to EGFR inhibitors (EGFRi) for patients with colorectal cancer (CRC) [7].

The RAS GTPases (KRAS, NRAS, and HRAS) are key intermediaries of the proliferation signals from EGFR. Oncogenic KRAS mutations are constitutively active in an EGFR independent manner and can activate the same effectors as wild-type RAS-GTPases. The three RAS GTPases all have mutation “hot-spots” at codons 12, 13, and 61 [8]. These mutations result in impaired GTPase activity and, most importantly, lack of sensitivity to Ras GTPase Activating Proteins (GAPs) that efficiently convert RAS-GTP to RAS-GDP [9]. Several different RAS GAPs have been identified [10]. The RAS GAP Neurofibromin (NF1) is known to play an important role in maintaining low levels of RAS-GTP in unstimulated cells, with loss-of-function NF1 mutations being common in cancer [11]. Oncogenic RAS insensitivity to GAP mediated inactivation follows from loss of key structural arrangements necessary for GTP hydrolysis [12]; although GAPs can typically bind to oncogenic mutant RAS proteins they are generally unable to promote hydrolysis as well as they can for wild-type RAS proteins [13].

Biochemical characterizations have revealed additional variations between individual hotspot RAS mutations [14]. For example, KRAS G13D (in which the thirteenth amino acid of KRAS, which is normally a Glycine, “G”, is replaced with an Aspartic Acid, “D”) has an elevated rate of spontaneous nucleotide exchange [14] and has been reported to be impaired at binding to NF1 [13].

As oncogenic KRAS mutants were constitutively active in an EGFR independent manner and can typically bind to the same downstream effectors as wild-type RAS proteins, it seemed logical to assume that all constitutively active KRAS mutations would cause resistance to EGFRi. Surprisingly, a retrospective analysis of phase three clinical trial data found that KRAS G13D was an exception to this rule [15]. A mechanism to explain why KRAS G13D behaves differently has been lacking, and the determination of this mechanism has been considered necessary for the retrospective studies to be used for patient management [16].

Two recent studies report investigations of this problem and present a mechanistic basis for this KRAS G13D-specific behavior [17, 18]. Both of these studies implicate NF1 as a critical variable that influences whether or not a KRAS mutant is sensitive to EGFRi. However, each study presents a different mechanism. The key difference in the two studies is whether EGFRi results in reduced GTP-bound mutant KRAS G13D (the Rabara et al mechanism [18]) or reduced wild-type (WT) NRAS and HRAS (the McFall et al mechanism [17]). Rabara et al posit that KRAS G13D retains sensitivity to NF1, and that inhibition of EGFR can thereby result in a reduction of active, GTP-bound, KRAS G13D through NF1 activity on KRAS G13D [18].

In contrast, McFall et al propose that the treatment of KRAS G13D CRC with EGFRi results in a reduction of NRAS-GTP and HRAS-GTP, with no change in active KRAS G13D [17]. Through mathematical modeling of the cellular processes that regulate RAS signaling, McFall et al revealed that NF1 can be competitively inhibited by KRAS mutants that bind NF1 well. This effectively reduces NF1 GAP activity on WT RAS-GTP and thereby promotes increased net activation of WT RAS proteins in an EGFR-independent manner [19]. In contrast, mathematical modeling revealed that KRAS G13D, which does not bind NF1 well, does not prevent NF1 from promoting GTP hydrolysis on WT RAS. This leaves WT RAS dependent upon other factors (such as EGFR) for activation [17].

Both groups presented experimental evidence that supports their distinct mechanisms. As uncertainty may cause clinicians to delay utilizing EGFRi on KRAS G13D CRC patients, it is important to resolve the apparent discrepancy between these studies.

## Results and discussion

### Cellular studies of NF1 activity on KRAS G13D

**Fig. 1:**
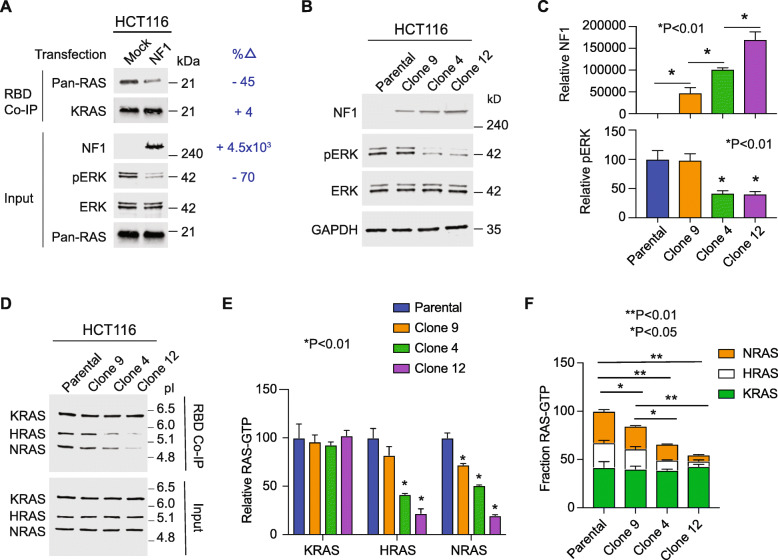
Evaluation of NF1 mediated hydrolysis of mutant and WT RAS. **a** Active Ras RBD pull down assays were performed on HCT-116 cells transfected to overexpress NF1. *n* = 1. **b** Immunoblots of HCT-116 clones that overexpress NF1 (left). *n* = 3. **c** Densitometry-based quantification of immunoblots, with means +/− SD, from three independent assays represented in (**b**). **d** RAS-GTP levels as measured by RBD pull-down followed by IEF to separate KRAS, HRAS, and NRAS in accordance with the isoelectric point (pI) of each. *n* = 3. **e** Densitometry based quantification of immunoblots, with means +/− SD, from three independent assays represented in (**d**). **f** Densitometry data from E, normalized to total RAS in the parental line. Indicated *P*-values are from One Way Anova followed by post-hoc Tukey’s test for multiple comparisons

Rabara et al described an experiment where they ectopically overexpressed NF1 in HCT-116 colorectal cancer cells (which are NF1 null and harbor a KRAS G13D mutation). They reported witnessing a reduction in KRAS-GTP upon NF1 overexpression [18]. This differs from the experiments we had performed, where we observed decreases in HRAS-GTP and NRAS-GTP, but not KRAS-GTP. To investigate, we performed the same experiment described by Rabara et al. Of critical importance, the methods of Rabara et al state that they utilized a KRAS antibody that was provided within a commercial Ras-Binding Domain (RBD) Ras-activation measurement assay; however, this kit does not provide a KRAS specific antibody. Instead, the kit supplies a pan-RAS antibody that detects HRAS, NRAS, and KRAS. We therefore performed this same experiment both with the RBD kit’s pan-RAS antibody and a separate KRAS-specific antibody to determine whether their experiment was detecting reductions in KRAS-GTP or reductions in total HRAS-GTP, NRAS-GTP, and KRAS-GTP. When we use the pan-RAS antibody we observe reduced total RAS-GTP in the NF1 transfected cells, however, when we probe with the KRAS-specific antibody we observe no change in KRAS-GTP (Fig. 1a). Based upon the described methods in Rabara et al, we propose that Rabara et al misinterpreted a reduction in HRAS-GTP and NRAS-GTP for a reduction in KRAS-GTP due to a misattribution of a pan-RAS antibody as a KRAS-specific antibody.

We considered the possibility that the level of NF1 expression varied between the two studies. To investigate whether increased levels of NF1 expression might result in reduced KRAS-GTP, we transduced HCT-116 cells with NF1 and generated three different clonal populations that each expressed NF1 at a different level. We combined isoelectric focusing (IEF) with the RBD Ras-activation assay to distinguish between KRAS-GTP, NRAS-GTP, and HRAS-GTP. We observed decreasing levels of NRAS-GTP and HRAS-GTP with increasing levels of NF1 expression without change in KRAS-GTP (Fig. 1b,c). This argues that NF1 cannot effectively reduce KRAS-GTP levels within cancer cells, even when highly overexpressed.

### Biophysical studies of NF1 activity on KRAS G13D

**Fig. 2:**
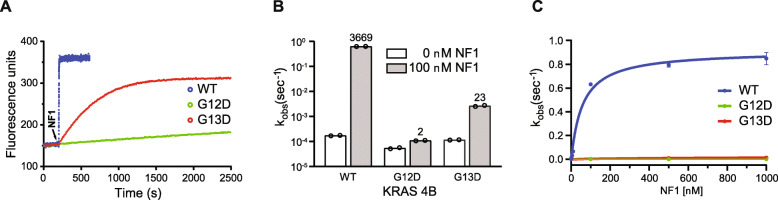
Biophysical evaluation of NF1 mediated hydrolysis on KRAS G13D. **a** Stimulation of the GTPase activity of KRAS 4B proteins by NF1–333. Reactions contained 1 μM KRAS 4B and 100 nM NF1–333. The arrow indicates the time point of NF1–333 addition. **b** The rates (k_obs_) of phosphate (P_i_) release. The numbers above the bars indicate the fold stimulation for the NF1-catalyzed reaction (100 nM NF1–333) over the unstimulated reaction (0 nM NF1–333) for each KRAS 4B protein (1 μM). *n* = 2. **c** the rate of P_i_ release as a function of NF1–333 concentration for each KRAS 4B protein. n = 2

Rabara et al also presented biophysical data that showed NF1–333 [20] converting KRAS G13D-GTP to KRAS G13D-GDP at a rate essentially equivalent to that measured for KRAS WT (WT k_obs_ = 0.0528 ± 0.0224 s^− 1^ vs. G13D k_obs_ = 0.0346 ± 0.0179 s^− 1^). We independently assessed NF1–333 GAP activity on KRAS G13D, KRAS G12D, and KRAS WT (Fig. 2a,b). Although we also detected NF1-stimulated GTP hydrolysis for KRAS G13D, the observed rate was ~ 0.4% of that measured for the GAP activity of NF1–333 on KRAS WT (WT k_obs_ = 0.6320 ± 0.0020 s^− 1^ vs. G13D k_obs_ = 0.0026 ± 0.0001 s^− 1^). These experiments were carried out using 1 μM KRAS-GTP and 100 nM NF1–333. We also performed these experiments for an extended range of NF1–333 concentrations (Fig. 2c). These data further highlight that GTP hydrolysis by NF1–333 was largely impaired for KRAS G13D relative to KRAS WT. Differences in experimental conditions may partially account for the discrepancy between our observed hydrolysis rates and those of Rabara et al, however, our cellular and biophysical data together argue that NF1 activity on KRAS G13D is not likely to be physiologically meaningful.

### BRET studies to evaluate KRAS mutant binding to NF1

**Fig. 3:**
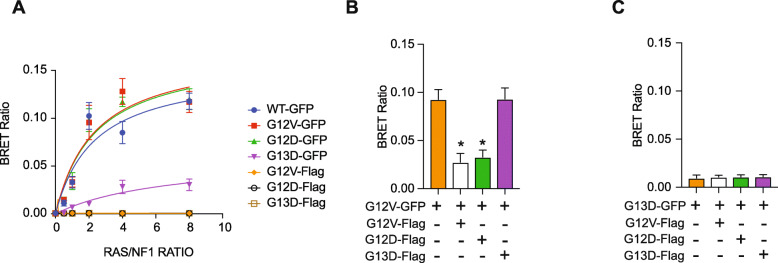
Competition studies show KRAS G13D is impaired at binding to NF1. **a** The mean BRET ratio for KRAS-GFP interactions with NF1-NanoLuc for increasing quantities of transfected KRAS constructs. KRAS-flag constructs were also included as a negative control. *n* = 3. **b** Assay using Flag-tagged G12V, G12D, or G13D KRAS to evaluate whether each mutant can compete with KRAS G12V-GFP to interact with NF1-Nano-Luc. n = 3. **c** Assay using Flag-tagged G12V, G12D, or G13D KRAS to evaluate whether each mutant can compete with KRAS G13D-GFP to interact with NF1-NanoLuc. n = 3

Previously, we utilized BRET to quantify the binding of GFP-tagged KRAS G12V, KRAS G13D, and KRAS WT with NF1, and we detected that KRAS G13D bound to NF1 (NF1-NanoLuc) less strongly than did KRAS G12V [17]. Reduced binding to NF1 is essential for the mechanism we propose [17, 21]. Here, we reproduce our published BRET study and include KRAS G12D to investigate a broader panel of KRAS mutants resistant to EGFRi. In this new study, we observe KRAS G12D binds to NF1 at a level comparable to KRAS G12V, consistent with our proposed mechanism (Fig. 3a).

We wished to further investigate the relative ability of KRAS G12V, G12D and G13D to bind to NF1. For this we adapted our BRET assay to evaluate whether Flag-tagged KRAS proteins, which do not produce a BRET signal with NF1-NanoLuc (Fig. 3a), can be used to compete with GFP-tagged KRAS G12V. We observed that both Flag-tagged KRAS G12V and KRAS G12D competed with KRAS G12V-GFP for binding to NF1, as evidenced by a reduction in the BRET signal, while Flag-tagged KRAS G13D could not (Fig. 3b). To investigate if the residual BRET signal observed in cells expressing KRAS G13D-GFP and NF1-NanoLuc is due to their specific interaction, we co-transfected Flag-tagged KRAS proteins. Flag-tagged KRAS G12V, KRAS G12D, or KRAS G13D did not reduce the measured BRET response suggesting the low-level BRET signal is due to non-specific interactions between GFP and NanoLuc (Fig. 3c).

## Conclusions

The study presented here proposes that the therapeutic response of KRAS G13D CRC to EGFRi follows from reductions to WT HRAS-GTP and WT NRAS-GTP. This work suggests that NF1 activity on KRAS G13D does not occur to a large extent, and that it is not occurring within cancer cells as no changes in KRAS-GTP were detected even at high levels of NF1 expression. Of note, much of the data presented by Rabara et al, including 1) the sensitivity of isogenic KRAS G13D SW48 cells to EGFRi relative to isogenic KRAS G12D SW48 cells and 2) the identification that NF1 expression is a critical variable that modulates sensitivity to EGFRi, are consistent with our model that reductions in WT HRAS-GTP and WT NRAS-GTP underlie the sensitivity of KRAS G13D colorectal cancer cells to EGFRi [17]. Additionally, co-mutations of NF1 with KRAS G13D and other mutations of G13 (ex. KRAS G13V and G13C) can be consistent with both mechanisms, as both suggest NF1 mutations result in elevated total RAS-GTP levels. Additional studies will be needed to determine whether other KRAS G13 mutations are impaired at binding to NF1, are sensitive to NF1 GAP activity, and/or whether co-mutation occurs due to another mechanism [22].

## Methods

### Cell culture

HCT116 cells were purchased from American Type Culture Collection (ATCC) were grown in McCoy’s 5A medium with FBS (10%), penicillin (100 U/ml), streptomycin (100 μg/ml), l-glutamine (2 mM) and incubated at 37 °C in 5% CO2 unless indicated otherwise in experimental methods.

### Cell transfection

Cells were plated in a 10 cm plate at a density of 6 × 10^6^ in antibiotic- free medium. Twenty-four hours later, cells were transfected with expression plasmids packaged in Lipofectamine 2000 (ThermoFisher) containing 5 μg of DNA following manufacturers protocol. Cell lysates were prepared 24 h post transfection for RAS activity analysis.

### RAS activity assay

Isolation of active RasGTP was performed using the Active Ras Pull- Down and Detection Kit (ThermoFisher) following the manufacturer’s protocol. Ras abundance was measured by Western blot. Western blot analysis of RBD pull-down lysates was performed with mouse anti-KRAS antibody (WH0003841, Sigma), mouse anti–pan-RAS antibody (1,862,335, ThermoFisher), mouse anti-GAPDH (sc-4772, Santa Cruz Biotechnology) mouse anti-phosphorylated ERK (675,506, Biolegend), Rat anti-ERK (686,902, Biolegend), mouse anti-NF1 (sc-376,886, Santa Cruz Biotechnology) .

### HCT116 NF1+ clone generation

293FT cells were used to generate lentiviral particles by transfection using Lipofectamine 2000 (Life Technologies Corporation). Packaging plasmids pMD2G, PMDLg/RRE, and pRSV/Rev. were co-transfected with pCDH NF1-NanoLuc C-term expression plasmid. Lentivirus containing supernatant was harvested at 48 and 72 h after transfection. HCT116 were plated in McCoy’s 5A media with heat-inactivated FBS (10%) and 2 mM l-glutamine 2 days before infection. For infection HCT116 were transduced with pCDH NF1-nanoLuc lentivirus with polybrene (8 μg/ml) for 10 h. The cells were washed, medium was replenished, and cells were incubated for 48 h in normal growth media. After this, cells were plated in 10 cm dishes at a density of 100 cells per plate in selection media (puromycin:1 μg/ml). When colonies formed (> 50 cells), colonies were extracted with cloning cylinders and expanded in individual 60 mm plates.

### RBD-IEF

HCT116 parental cells and NF1-clones were cultured in 10 cm culture dishes. Medium was removed, and cells were washed with ice- cold tris-buffered saline. Cells were scraped in 1 ml of lysis wash buffer [25 mM tris-HCl (pH 7.2), 150 mM NaCl, 5 mM MgCl2, 1% NP-40, and 5% glycerol]. Cells were lysed on ice and vortexed every 10 s. Cell lysates were subjugated to RBD coimmunoprecipitation as previously described above. RBD coimmunoprecipitation product was resolved by SDS–polyacrylamide gel electrophoresis in a 12% polyacrylamide gel. Bands were excised from the 21-kDa region of the gel. Gel products were liquified at 95 °C for 5 min. Protein was extracted and purified using the ReadyPrep 2-D Cleanup Kit (BioRad) following the manufacturer’s protocol. Protein samples were added to 50% glycerol loading buffer and incubated at room temperature for 20 min. Samples were resolved on Criterion Bio-Lyte IEF Gel with a 3 to 10 pH range (Bio-Rad Laboratories). Gels were run at the following power conditions with constant voltage: 100 V for 60 min, 250 V for 60 min, and 500 V for 30 min in a stepwise fashion with a total run time of 150 min. The IEF gel was then soaked in 5% SDS buffer for 24 h with gentle rocking at 4 °C. Protein was electrophoretically transferred to PVDF membranes (Millipore Corporation) for 1 h at a constant 25 V. The PVDF blots were probed with the anti–pan-RAS primary antibody from the Active Ras Pull-Down and Detection Kit (ThermoFisher) and the anti-mouse DyLight 800 fluorophore-conjugated secondary antibody (Invitrogen). The protein bands were visualized using the Licor CLx Odyssey imaging station (Licor Biosystems).

### Protein expression and purification

A human KRAS 4B DNA fragment encoding residues 1–185 (C185S) was subcloned into pProEx HTb in frame with an N-terminal His_6_-tag for protein expression. Site-directed mutagenesis was subsequently performed to generate KRAS 4B G12D and G13D expression constructs. A pQlinkHG-k NF1 expression construct encoding NF1 residues 1197–1528 (NF1–333) fused to an N-terminal His6/GST-tag was a generous gift from S. Campbell (University of North Carolina, Chapel Hill). Protein expression was carried out using Rosetta2 BL21(DE3) cells (Novagen). Bacterial cultures were grown in TB/AMP/CHL at 37 °C to an OD_600_ of 0.6 and then induced with 0.1 mM IPTG for ~ 18 h with continuous shaking at 22 °C. Bacterial cell pellets were resuspended in buffer N1 (20 mM Tris pH 8.0, 300 mM NaCl, 5% glycerol and 10 mM imidazole) then lysed using an Emulsiflex C5 homogenizer (Avestin). Lysates were clarified by centrifugation at 40,000 x g at 4 °C for 30 min and loaded onto a 5 ml HisTrap column (Cytiva) equilibrated in N1, then protein was eluted in N1 containing 300 mM imidazole. Eluted proteins were incubated with the TEV protease overnight to release the N-terminal affinity tags while being dialyzed in SEC buffer (20 mM Tris pH 8.0, 200 mM NaCl, 2 mM DTT and 5% glycerol) and subsequently loaded onto an HiLoad Superdex 200 pg size exclusion column (Cytiva; 16/600) equilibrated in SEC buffer. Fractions containing purified KRAS4B or NF1–333 proteins were pooled, concentrated in a 10 K MWCO Spin-X UF (Corning) 20 ml concentrator, snap frozen in liquid N2 and stored at − 80 °C.

### Biophysical characterization of NF1 activity

Stimulation of the GTPase activity of KRAS 4B proteins (residues 1–185) by NF1–333 [20] was monitored using the fluorescent phosphate sensor, MDCC-PBP [23] and a Varian Cary Eclipse fluorescence spectrophotometer. Reactions were carried out using 1 μM KRAS-GTP, 0–1000 nM NF1–333 and 1.5 μM MDCC-PBP in a buffer containing 20 mM Tris-HCl pH 7.6, 50 mM NaCl and 2 mM MgCl_2_. Rates (k_obs_) of phosphate (P_i_) release were determined by fitting the fluorescence data as one phase associations in GraphPad Prism.

### Bioluminescence resonance energy transfer (BRET) assay

Human embryonic kidney (HEK)–293 T cells were grown in DMEM/10% FBS without antibiotic. Cells were seeded at 5 × 10^3^ cells per well in a 96-well white opaque Perkin Elmer microplate. Twenty-four hours after seeding, cells were cotransfected with a constant concentration of 0.1 μg of NF1-NanoLuc pcDNA expression plasmid and in- creasing concentrations of RAS-EGFP pcDNA or RAS-FLAG pcDNA expression plasmid (0, 0.05, 0.1, 0.2, 0.4, and 0.8) with 0.25 μl of Lipofectamine 2000 per well following the manufacturer’s protocol (ThermoFisher). Twenty-four hours later, medium was aspirated from each well and 25 μl of NanoGlo Live Cell Reagent was added to each well per the manufacturer’s protocol (Promega). Plates were placed on orbital shaker for 1 min at 300 rpm. After incubation, the plate was read on the Tecan Infinite M200 PRO with LumiColor Dual Setting with an integration time of 1000 ms. BRET ratio was calculated from the dual emission readings. BRET ratio was plotted as a function of the RAS-GFP/NF1-NanoLuc plasmid ratio. Competitive BRET was performed by transfecting equal (0.2 μg) amounts of donor (RAS- EGFP pcDNA) and competing (RAS-FLAG pcDNA) plasmids with 0.1 μg of NF1-nanoLuc pcDNA plasmid. BRET assays were repeated three times, each with eight biological replicates.

## Data Availability

All data obtained and/or analyzed within the current study are available upon reasonable request from the corresponding author.

## Abbreviations

CRC: colorectal cancer
EGFR: Epidermal Growth Factor Receptor
EGFRi: EGFR inhibitor
G12D: glycine at codon 12 replaced with aspartic acid
G12V: glycine at codon 12 replaced with valine
G13D: glycine at codon 13 replaced with aspartic acid
GAP: GTPase Activating Protein
GFP: Green fluorescent protein
IEF: Isoelectric focusing
PTB: Protein Tyrosine Binding
RBD: Ras Binding Domain
SH2: Src Homology 2
WT: wild-type

## References

[1] Hynes NE, Lane HA (2005). ERBB receptors and cancer: the complexity of targeted inhibitors. Nat Rev Cancer.

[2] Citri A, Yarden Y (2006). EGF-ERBB signalling: towards the systems level. Nat Rev Mol Cell Biol.

[3] Jones RB, Gordus A, Krall JA, MacBeath G (2006). A quantitative protein interaction network for the ErbB receptors using protein microarrays. Nature.

[4] Yarden Y, Sliwkowski MX (2001). Untangling the ErbB signalling network. Nat Rev Mol Cell Biol.

[5] Shepherd FA (2005). Erlotinib in previously treated non-small-cell lung cancer. N Engl J Med.

[6] Bollag G (2012). Vemurafenib: the first drug approved for BRAF-mutant cancer. Nat Rev Drug Discov.

[7] Karapetis CS (2008). K-ras mutations and benefit from cetuximab in advanced colorectal cancer. N Engl J Med.

[8] Prior IA, Lewis PD, Mattos C (2012). A comprehensive survey of Ras mutations in cancer. Cancer Res.

[9] Stephen AG, Esposito D, Bagni RK, McCormick F (2014). Dragging ras back in the ring. Cancer Cell.

[10] Vigil D, Cherfils J, Rossman KL, Der CJ (2010). Ras superfamily GEFs and GAPs: validated and tractable targets for cancer therapy?. Nat Rev Cancer.

[11] Sherekar M (2020). Biochemical and structural analyses reveal that the tumor suppressor neurofibromin (NF1) forms a high-affinity dimer. J Biol Chem.

[12] Scheffzek K (1997). The Ras-RasGAP complex: structural basis for GTPase activation and its loss in oncogenic Ras mutants. Science.

[13] Ahmadian MR, Mittal R, Hall A, Wittinghofer A (1997). Aluminum fluoride associates with the small guanine nucleotide binding proteins. FEBS Lett.

[14] Hunter JC (2015). Biochemical and structural analysis of common Cancer-associated KRAS mutations. Mol Cancer Res.

[15] De Roock W (2010). Association of KRAS p.G13D mutation with outcome in patients with chemotherapy-refractory metastatic colorectal cancer treated with cetuximab. JAMA.

[16] Morelli MP, Kopetz S (2012). Hurdles and complexities of codon 13 KRAS mutations. J Clin Oncol.

[17] McFall T, Diedrich JK, Mengistu M, Littlechild SL, Paskvan KV, Sisk-Hackworth L, Moresco JJ, Shaw AS, Stites EC. A systems mechanism for KRAS mutant allele–specific responses to targeted therapy. Sci Signal. 2019;12:eaaw8288. 10.1126/scisignal.aaw8288 PMC6864030 31551296

[18] Rabara D (2019). KRAS G13D sensitivity to neurofibromin-mediated GTP hydrolysis. Proc Natl Acad Sci U S A.

[19] Stites EC, Trampont PC, Ma Z, Ravichandran KS (2007). Network analysis of oncogenic Ras activation in cancer. Science.

[20] Scheffzek K (1998). Structural analysis of the GAP-related domain from neurofibromin and its implications. EMBO J.

[21] McFall T, Stites EC (2020). A mechanism for the response of KRAS(G13D) expressing colorectal cancers to EGFR inhibitors. Mol Cell Oncol.

[22] E. C. Stites, P. C. Trampont, L. B. Haney, S. F. Walk, K. S. Ravichandran, Cooperation between Noncanonical Ras Network Mutations Cell Rep 10.1016/j.celrep.2014.12.035 (2015). 10.1016/j.celrep.2015.01.048 30849858

[23] Brune M, Hunter JL, Corrie JE, Webb MR (1994). Direct, real-time measurement of rapid inorganic phosphate release using a novel fluorescent probe and its application to actomyosin subfragment 1 ATPase. Biochemistry.

